# Efficacy and safety of baricitinib for the treatment of hospitalized adults with COVID-19: a systematic review and meta-analysis

**DOI:** 10.1186/s40001-023-01403-0

**Published:** 2023-11-21

**Authors:** Jing Sun, Shufang Wang, Xin Ma, Qingqing Wei, Yujuan Peng, Ying Bai, Guobin Miao, Chang Meng, Peng Liu

**Affiliations:** 1 Department of Critical Care Medicine, Emergency General Hospital, Beijing, China; 2 Department of Emergency, Emergency General Hospital, Xibahe South Road 29, Chaoyang District, Beijing, 100028 People’s Republic of China; 3 Department of Critical Care Medicine, Affiliated Hospital of Hebei University, Baoding, China; 4 Department of Cardiology, Ordos School of Clinical Medicine, Ordos Central Hospital, Inner Mongolia Medical University, 23 Yijin Huoluo West Street, Dongsheng District, Inner Mongolia, 017000 People’s Republic of China

**Keywords:** Baricitinib, COVID-19, Efficacy, Safety

## Abstract

**Objectives:**

Several clinical trials have evaluated the efficacy and safety of baricitinib in COVID-19 patients. Recently, there have been reports on critical patients, which are different from previous research results. The meta-analysis was performed to investigate the effects of baricitinib in COVID-19, by pooling data from all clinically randomized controlled trials (RCTs) available to increase power to testify.

**Methods:**

Studies were searched in PubMed, Embase, and Cochrane Library databases on January 31, 2023. We performed a meta-analysis to estimate the efficacy and safety of baricitinib for the treatment of hospitalized adults with COVID-19. This study is registered with INPLASY, number 202310086.

**Results:**

A total of 3010 patients were included in our analyses. All included studies were randomized controlled trials or prospective study. There was no difference in 14-day mortality between the two groups [OR 0.23 (95% CI 0.03–1.84), *I*^2^ = 72%, *P* = 0.17]. In subgroup analyses we found that baricitinib did not seem to improve significantly in 24-day mortality critically ill patients [OR 0.60 (95% CI 0.35–1.02), *I*^2^ = 0%, *P* = 0.06]. Fortunately, baricitinib have led to faster recovery and shorter hospital stays for COVID-19 patients. There were no difference in infections and infestations, major adverse cardiovascular events, deep vein thrombosis and pulmonary embolism.

**Conclusions:**

Baricitinib did not increase the incidence of adverse reactions. At the same time, we can find that it reduces the mortality of COVID-19 patients, not including the critically ill.

**Supplementary Information:**

The online version contains supplementary material available at 10.1186/s40001-023-01403-0.

## Introduction

Many COVID-19 remains an important cause of death in recent years, especially among unvaccinated people with comorbidities or the elderly. A large number of literatures have reported that SARS-CoV-2 infection is often accompanied by excessive inflammation, which may lead to multiple organ dysfunction and even death [1–3]. People are constantly seeking for better drugs to improve patient mortality, including Baricitinib [4]. Barisinib is an oral Janus kinase (JAK) 1/2 inhibitor that was previously approved by the European Medicines Agency (EMA) for several chronic autoimmune diseases [5].

Studies have found that barisinib can reduce inflammatory storms, and serological examination showed that the application of the drug reduced cytokines and biomarkers related to the pathophysiology of COVID-19 in patients [6–8]. Later, World Health Organization (WHO) guidelines recommended the use of baricitinib, a Jak 1,2 inhibitor, for hospitalized COVID-19 patients receiving corticosteroid treatment. However, at that time, the relevant clinical evidence was relatively limited, so WHO recommended initiation of treatment “depending on availability,” as well as “clinical and contextual factors” [9].

In the past, five clinical studies [4, 6, 10–12] have compared the efficacy and safety endpoint of baricitinib and placebo for COVID-19 patients. We analyzed 14-day mortality, 28-day mortality, recovery and shorter hospital stays as efficacy endpoints of the study. The safety outcomes include infections and infestations, major adverse cardiovascular events, deep vein thrombosis and pulmonary embolism. Although all of these studies included patients with COVID-19, the severity of the groups included in different studies varied, and their conclusions were inconsistent. While the novel coronavirus is still prevalent today, many countries are facing multiple rounds of virus impact. Our study systematically evaluated the mortality, length of stay and related adverse events of hospitalized patients with COVID-19 after the application of basitinib, which will provide certain guidance for clinical practice.

## Methods

We carried out the meta-analysis in accordance with the Preferred Reporting Items for Systematic Reviews and Meta-analyses (PRISMA) guidelines [13]. Our protocol was registered on the International Platform of Registered Systematic Review and Meta-analysis Protocols database (Inplasy protocol: INPLASY202310086), and is available in full on inplasy.com (https://inplasy.com/inplasy-2023-1-0086). Ethics approval was not required for our work.

### Search strategy

Three independent researchers (Jing Sun, Shufang Wang and Xin Ma) conducted extensive electronic searches for relevant articles published on Jan 31, 2023. The database includes PubMed, Embase and the Cochrane database. Manually select relevant randomized controlled trial. The search strategy of the literature was shown in the supplement (Additional file 1: Table S1).

### Inclusion and exclusion

EndNote (X9 version) software is selected for document management, two investigators independently evaluated the eligibility of the identified items. The title and summary are filtered for the first time, and qualified articles are reserved for full-text review. The included studies were randomized controlled trials. Inclusion criteria for studies meeting the following requirements include: (1) Patients of hospitalized adults with COVID-19. (2) Treatment with baricitinib or placebo or conventional therapy. (3) Outcomes Indicators: Death from any cause/Duration of hospitalization/ Median time to recovery/ lnfections and infestations/Major adverse cardiovascular events (MACEs)/Pulmonary embolism (PE)/Deep vein thrombosis(DVT), including one. We excluded animal testing, studies enrolling patients < 18 years old, and there was not enough data to extract, such as the summary of some meetings, literature materials such as review and pharmacological introduction. Documents that are not consistent with the content of this study will also be excluded. We contacted the authors if associated data from their studies were required.

### Bias & quality assessment

The two researchers independently evaluated, preliminarily selected and checked the literature data according to the unified and standardized method, and included them in the literature in strict accordance with the admission and exclusion criteria, and then collected information. Evaluate the quality of selected articles according to the quality evaluation standard of Cochrane Reviewer Handbook 5.1.0 [14].

### Data synthesis and analysis

Revman5.3 were used for meta-analysis. Data which met homogeneity (*P* > 0.10 and I2 ≤ 50%) through heterogeneity test were meta-analyzed using fixed effect model. If homogeneity (*P* ≤ 0.10 or I2 > 50%) was not met, and heterogeneity cannot be ruled out, random effect model can be used to combine effects [15]. While it should be noted that sensitivity analysis and subgroup analysis should be considered for this type of analysis data. Results were expressed as odds ratio (OR) with a 95% confidence interval (CI) with discontinuous outcomes. For the continuous outcomes, mean differences (MD) and 95% CIs were estimated as effective. Some included RCTs reported median as the measure of treatment effect, with interquartile range (IQR). We estimated the mean from median and standard deviations (SD) from IQR using the methods described in the previous studies [16]. A p-value < 0.05 was considered statistically significant.

## Results

**Fig. 1:**
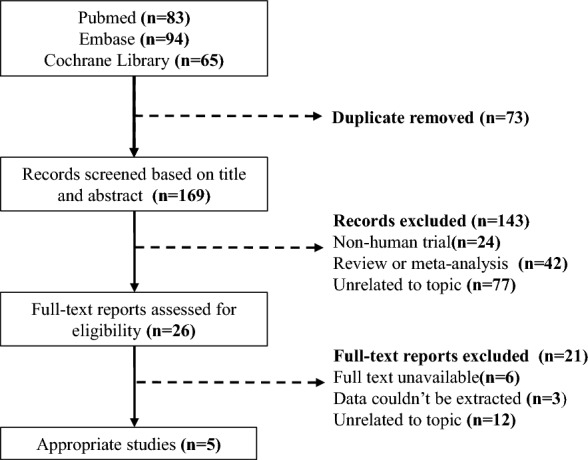
The flow chart of the search and study selection process

The flowchart (Fig. 1) summarizes the search and study selection process. A total of 242 related literatures were retrieved, of which 73 were excluded due to duplication. 143 studies were also excluded after reading the titles and abstracts. The remaining 26 studies were assessed by reading the full texts. Data from 5 trails evaluating the Efficacy and safety of baricitinib for the treatment of hospitalized adults with COVID-19 were included.

The main features of included trials are presented in Table 1. A total of 3010 patients were included in our analyses. All included studies were randomized controlled trials or prospective study. All of the studies were comparing the efficacy and safety of baricitinib for the treatment of hospitalised adults with COVID-19. The first three of the five studies in Table 1 are for hospitalized patients who have all been diagnosed with COVID-19, and the last two are for critically ill COVID-19 patients who have severe oxygenation disorder or receive mechanical ventilation/extracorporeal membrane oxygenation. No differences were observed in terms of proportion of patients lost to follow up across trials.

**Table 1:** Design and outcomes of the studies included in the meta-analysis

Num	Author/ Year	Design	Intervention assignments		Participants				Outcomes
			Baricitinib	Control	Sample size, n	Mean age, years(B/C)	Male:Female, (B/C)	Time of medication	
1	Bronte/2020	PS, MC	4mg bid for 2 days, followed by 4mg qd	conventional therapy	76	68/77.5	7:13/31:25	7 days	All cause deaths; Incidence of ARDS; Duration of hospitalization
2	Kalil/2021	RCTs,MC	4-mg qd	Placebo	1033	55/55.8	319:196/333:185	14 days or until hospital discharge	14-day mortality, 28-day mortality Median time to recovery
3	Marconi/2021	RCTs,MC	4-mg qd	Placebo	1525	57.8/57.5	490:274/473:288	14 days	28-day mortality; Median time to recovery; Duration of hospitalisation
4*	Ely/2022	RCTs,MC	4-mg qd	Placebo	101	58.4/58.8	25:26/ 30:20	14 days	28-day mortality;Treatment-emergent infection; DVT; PE; MACEs
5*	Trøseid/2023	RCTs,MC	4-mg qd	Placebo	275	59/60	112:27/99:37	14 days	28-day mortality; 60-day mortality; Infections and infestations; DVT; PE; MACEs

*ARDS* Acute respiratory distress syndrome; *B/C* baricitinib group/ control group; *Bid* twice daily; *DVT* deep vein thrombosis; *MACEs* Major adverse cardiovascular events; *MC* Multicenter; *PE* pulmonary embolism; *PS* prospective study; *Qd* Once a day; *RCTs* randomized clinical trials

^*^Severe or critical COVID-19

**Fig. 2:**
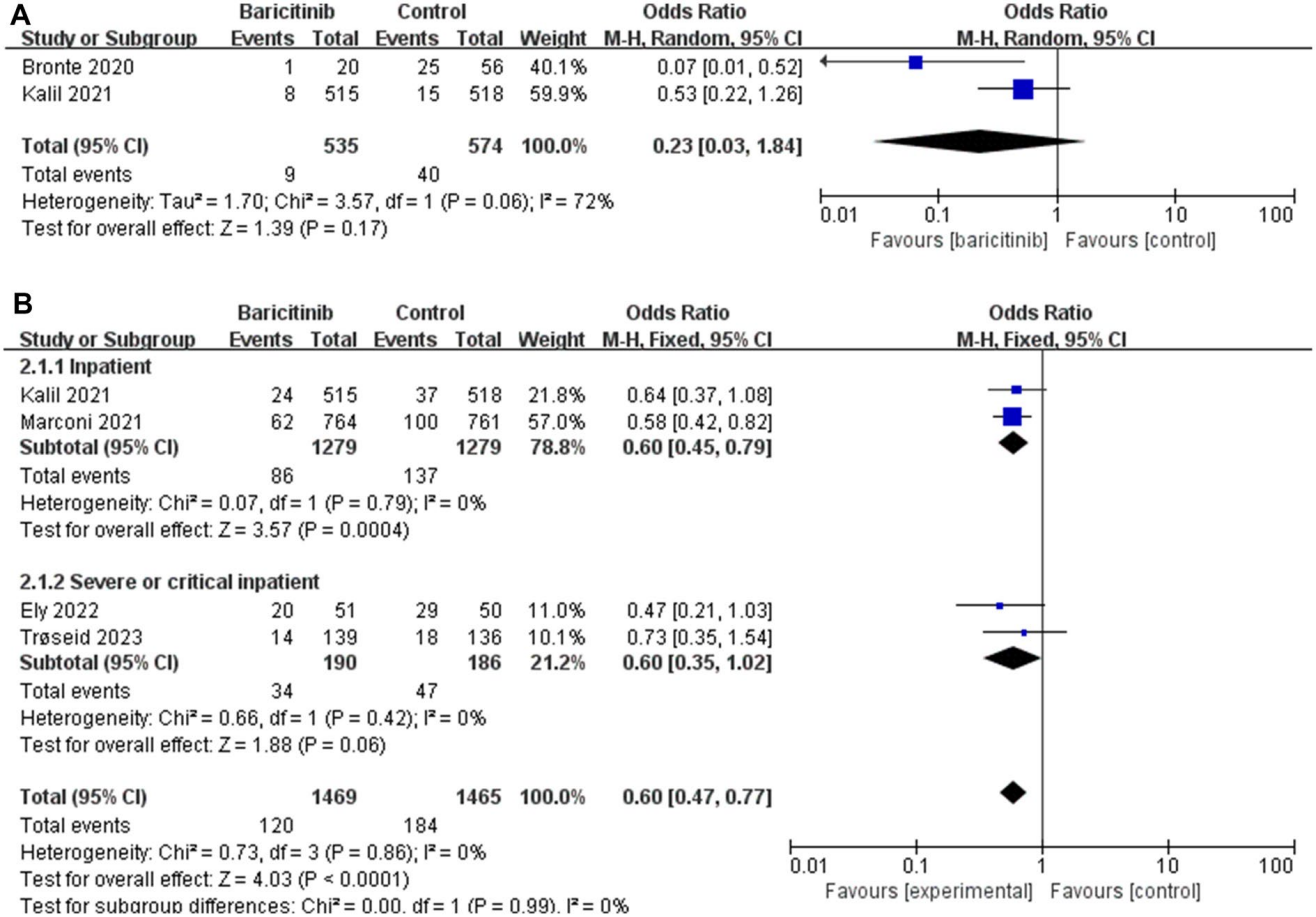
**a** The efficacy outcomes of 14-day mortality. **b** The efficacy outcomes of 28-day mortality

**Fig. 3:**
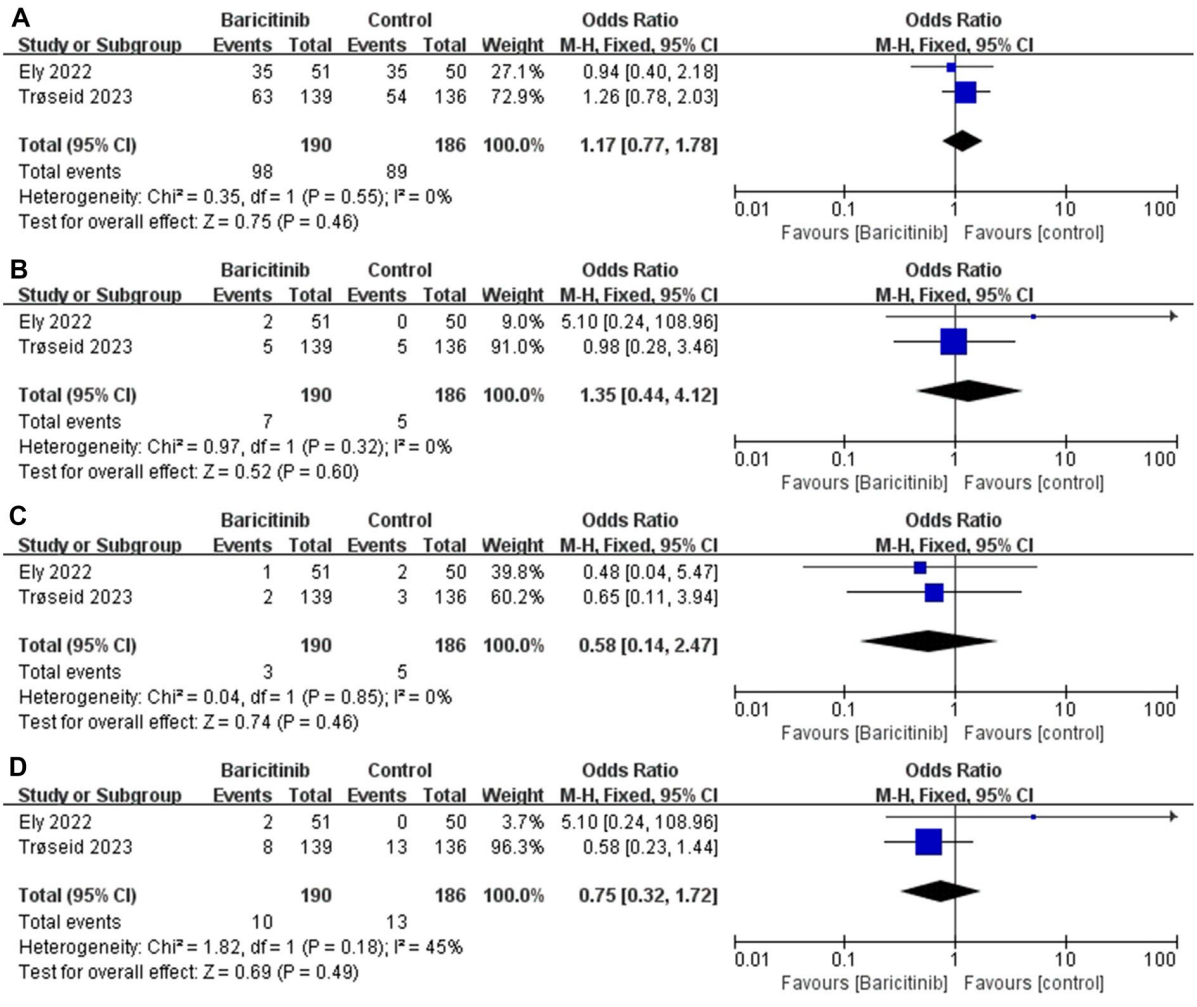
The safety outcomes of infections and infestations (**a**), major adverse cardiovascular events (**b**), deep vein thrombosis (**c**) and pulmonary embolism (**d**)

The efficacy outcomes are summarized in Fig. 2A, B, C and D in the Additional file 2: Figure S2AB). There was no difference in 14-day mortality (A) between the two groups [OR 0.23 (95% CI 0.03–1.84), *I*^2^ = 72%, *P* = 0.17]. Four studies reported 28-day mortality (B) outcomes in which baricitinib improved patient outcomes [OR 0.60 (95% CI 0.47–0.77), *I*^2^ = 0%, *P* < 0.0001]. To further analyze the causes, we then performed a subgroup analysis according to disease severity. In subgroup analyses we saw that baricitinib did not seem to improve significantly in critically ill patients [OR 0.60 [95% CI 0.35–1.02], *I*^2^ = 0%, *P* = 0.06]. Fortunately, baricitinib have led to faster recovery (D) and shorter hospital stays(C) for COVID-19 patients [MD = − 1.00 (95% CI − 1.12 to − 0.88), *I*^2^ = 0%, *P* < 0.0001; MD = − 0.80 (95% CI − 0.84 to − 0.76), *I*^2^ = 0%, *P* < 0.0001]. Due to the limited number of reports on the results of the current study, no further analysis is being conducted here. Based on previous experience, it is speculated that this may also be related to the severity of the disease. The safety outcomes are summarized in Fig. 3. There were no difference in infections and infestations (a), major adverse cardiovascular events (b), deep vein thrombosis(c) and pulmonary embolism (d). However, these results are based on the results of two randomized controlled trials conducted in patients with critically ill COVID-19.

We use Revman to investigate the influence of a single study on the overall pooled estimate of each predefined outcome. We found that the removal of any one study would not affect the following results. The results of the risk of bias assessment of these trials are summarized in the Additional file 1: Figure S1. Three studies were considered at low risk for overall risk of bias.

## Discussion

This outbreak initially attracted people's attention as an unusual viral pneumonia, and atypical upper respiratory pneumonia has been the main characteristic disease severity of this outbreak so far [17]. Bronchoalveolar lavage fluid was derived from macrophages with high levels of chemokines secreted by severe pneumonia [18]. Postmortem lung tissue analysis of COVID-19 patients with severe pneumonia also found excessive immune cell infiltration [19]. Baricitinib, inhibitors of Janus kinase (JAK)-1 and JAK-2, plays an important role in the regulation of immune response. COVID-19 is still circulating, and different mutated strains are still affecting our lives nowadays. A more detailed mechanism of action may be the direction of future research, including mixing with other drugs. Our meta-analysis system evaluated the efficacy and safety of baricitinib, which provides a good description for future clinical applications.

This study systematically evaluated the efficacy and safety of basitinib in the treatment of COVID-19 patients by including 5 high-quality studies. It is a meta-analysis with the largest sample size of baricitinib and a high level of evidence. In the analysis of mortality, we adopted 14-day mortality and 28-day mortality. The results showed that baricitinib application improved 28-day mortality in general hospitalized patients, but did not improve 14-day mortality in hospitalized patients or 28-day mortality in critically ill patients. Based on the current evidence, we analyzed that the lack of improvement in 14-day mortality may be related to the small number of studies at present. However, in the description of 28-day mortality, we can see that baricitinib reduces the mortality of hospitalized patients with non-severe COVID-19, which also suggests that the importance of baricitinib in combination with other treatment options for critically ill patients. There have also been studies claiming that the risk/benefit ratio of baricitinib in patients with severe/critical COVID-19 may vary depending on the immune status of SARS-CoV-2, and that potential host factors such as comorbidibility, older age and possible immune response [20] may contribute to this difference, which is worth further analysis and research in the future.

Our study, which pooled existing high-quality studies, has clear advantages, particularly in terms of mortality, and conducted a subgroup analysis of patients who were not at risk, revealing the different effects of the drug in different patients. And the safety of drugs in critically ill patients was analyzed. It provides a strong guiding value for clinic. Of course, this study also has some limitations. The current number of studies is relatively small, and more RCTs are needed to support it in the future.

## Conclusions

Baricitinib shortens the length of hospital stays and reduces the mortality of non-severe COVID-19 patients. It should be noted that the effect of drugs on the mortality of critical ill patients is not significant.

### Supplementary Information

**Additional file 1: Table S1.** Search strategy.** Figure S1.** Risk of bias graph.

**Additional file 2: Figure S2A.** The efficacy outcomes of hospital stays. **B** The efficacy outcomes of recovery.

## Data Availability

Data sets are available on request from the corresponding author.
