# Supplementary material for: Efficacy and safety of baricitinib for the treatment of hospitalized adults with COVID-19: a systematic review and meta-analysis
Source: Eur J Med Res. 2023 Nov 21;28:536. doi: 10.1186/s40001-023-01403-0 (PMC10661565; doi:10.1186/s40001-023-01403-0)

**Table S1. Search strategy.**

| Electronic database | Search strategy |
| --- | --- |
| PubMed (NCBI) | ("COVID 19"[Title/Abstract] OR "2019-nCoV Infection"[Title/Abstract] OR "SARS-CoV-2 Infection"[Title/Abstract] OR "COVID-19 Virus Infection"[Title/Abstract] OR "COVID-19 Virus Disease"[Title/Abstract] OR "2019 nCoV Disease"[Title/Abstract]) AND ("Baricitinib"[Title/Abstract] OR "INCB 028050"[Title/Abstract] OR "BARICITINIB"[Title/Abstract] OR "LY 3009104"[Title/Abstract]) AND ("randomized controlled trial" OR "randomized" OR "placebo" OR "randomly" OR "trial" [Title/Abstract]) |
| Embase | #1 (‘COVID 19’:ab,ti OR ‘2019-nCoV Infection’:ab,ti OR ‘SARS-CoV-2 Infection’:ab,ti OR ‘COVID-19 Virus Infection’:ab,ti OR ‘COVID-19 Virus Disease’:ab,ti OR ‘COVID-19 Virus Disease’:ab,ti)  #2 (‘Baricitinib’:ab,ti OR ‘INCB 028050’:ab,ti OR 'BARICITINIB':ab,ti OR 'LY 3009104':ab,ti)  #3 (‘randomized controlled trial’:ab,ti OR ‘randomized’:ab,ti OR ‘placebo’:ab,ti OR ‘randomly’:ab,ti OR ‘trial’:ab,ti)  #4 #1 AND #2 AND #3 |
| Cochrane | (“COVID 19” OR “2019-nCoV Infection” OR “SARS-CoV-2 Infection” OR “COVID-19 Virus Infection” OR “COVID-19 Virus Disease” OR “COVID-19 Virus Disease”) AND (“Baricitinib” OR “INCB 028050” OR “BARICITINIB” OR “LY 3009104”) |

**Figure S1. Risk of bias graph**


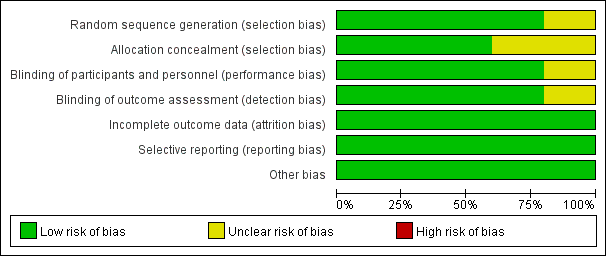

Supplement: Supplementary file 1 — Additional file 1: Table S1. Search strategy. Figure S1. Risk of bias graph. [file 40001_2023_1403_MOESM1_ESM.docx]
