# Supplementary figures and images for: Efficacy and safety of baricitinib for the treatment of hospitalized adults with COVID-19: a systematic review and meta-analysis
Source: Eur J Med Res. 2023 Nov 21;28:536. doi: 10.1186/s40001-023-01403-0 (PMC10661565; doi:10.1186/s40001-023-01403-0)

Figure 2A


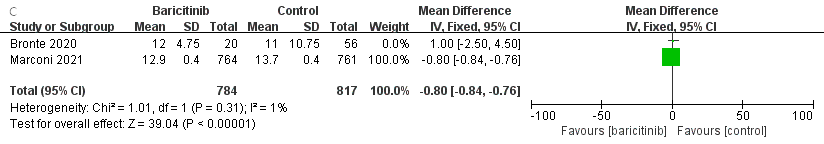


Figure 2B


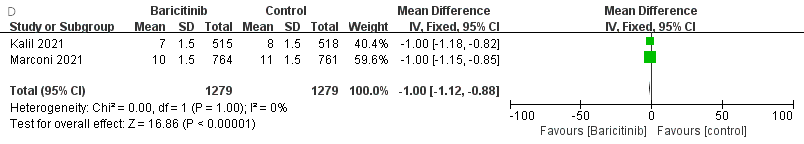

Supplement: Supplementary file 2 — Additional file 2: Figure S2A. The efficacy outcomes of hospital stays. B The efficacy outcomes of recovery. [file 40001_2023_1403_MOESM2_ESM.docx]
